# Analytical and numerical properties of an extended angiogenesis PDEs model

**DOI:** 10.1007/s00285-025-02293-y

**Published:** 2025-10-22

**Authors:** Pasquale De Luca, Livia Marcellino

**Affiliations:** Department of Science and Technology, Parthenope University of Naples, Centro Direzionale C4, 80143 Naples, Italy

**Keywords:** Angiogenesis, Partial differential equations, Numerical methods for PDEs, Convergence analysis

## Abstract

This paper presents an extended mathematical model for tumor angiogenesis incorporating oxygen dynamics as a main regulator. We enhance a five-component PDE system describing endothelial cells, proteases, inhibitors, extracellular matrix, and oxygen concentration, with a focus on their spatiotemporal interactions. We establish existence, uniqueness, and boundedness of solutions through a mathematical analysis. A numerical scheme using method of lines and fourth-order Runge-Kutta methods is developed, with proven stability constraints and convergence properties. Numerical experiments demonstrate biologically plausible vascular formation with oxygen-mediated regulation.

## Introduction

Angiogenesis, the formation of new blood vessels from pre-existing vasculature, plays a main role in physiological processes such as wound healing, embryonic development, and tumor growth. Understanding the mechanisms governing angiogenesis is fundamental for advancing treatments in regenerative medicine and cancer therapy. Mathematical modeling provides a powerful tool to investigate the complex interactions of endothelial cells, biochemical signals, and mechanical factors involved in angiogenesis. Among the various approaches, partial differential equations (PDEs) have been widely employed to describe spatial and temporal dynamics, offering insights into the behavior of vascular networks under different conditions. Existing models of angiogenesis often rely on reaction-diffusion systems and chemotactic mechanisms to simulate endothelial cell migration and vessel formation. While these models have been instrumental in capturing essential angiogenic features, they often simplify the influence of additional biological and physical factors. Early continuum models by Anderson and Chaplain (1998) established the foundation for PDE-based angiogenesis modeling by incorporating endothelial cell random motility, chemotaxis, and haptotaxis. In Mantzaris et al. (2004) the authors extended these models by including cell proliferation and death, while in McDougall et al. (2006) incorporated blood flow and vascular remodeling. More recent developments have significantly expanded the scope and complexity of angiogenesis models. The authors, in Xu et al. (2016), developed a multi-scale model integrating cellular, molecular, and tissue-level processes with a particular focus on the role of matrix metalloproteinases. For instance, in Vavourakis et al. (2017), authors proposed a hybrid continuum-discrete approach that couples blood flow with angiogenic sprouting, capturing the dynamic interplay between mechanical forces and chemical signals. The work of Vilanova et al. (2018) introduced a mechanochemical model that explicitly accounts for the interactions between cell-generated forces and the extracellular matrix during sprouting angiogenesis. The role of oxygen as a key regulator in angiogenesis has received increasing attention. Oxygen gradients not only trigger the initial angiogenic response but also guide vessel formation and maturation. A multi-phase model incorporating oxygen transport was developed by Grogan et al. (2017), demonstrating how hypoxia-driven vascular growth affects tumor progression. Similarly, a modeling framework capturing feedback loops was presented by Stepanova et al. (2021), including oxygen delivery, vessel formation, and tumor growth. Recent work by Hormuth et al. (2021) introduced a detailed model accounting for the dynamic interplay between oxygen tension and endothelial cell phenotype, showing how oxygen levels regulate the switch between tip and stalk cell behaviors. Moreover, computational approaches have advanced significantly, allowing for more realistic simulations. The author of Botti et al. (2010) implemented adaptive mesh refinement techniques to efficiently resolve the fine structures of growing vascular networks. The work (Lee et al. 2024) leveraged machine learning algorithms to optimize parameter estimation in angiogenesis models, improving their predictive capabilities. Advanced numerical techniques for evolving PDEs on complex domains have been explored in Sokolov et al. (2015) and (Conte et al. 2023), providing insights relevant to angiogenesis modeling on time-varying geometries. Computational approaches for coupled transport phenomena, as detailed in Hayat et al. (2023), offer valuable perspectives for oxygen diffusion modeling in biological tissues. Recent developments in numerical methods for integral-differential equations (Abduzhabbarov et al. 2021) may prove beneficial for future model extensions incorporating non-local interactions.

In this work, we extend a previous mathematical framework from Kevrekidis et al. (2005) and studied in De Luca and Marcellino (2025) by incorporating the oxygen concentration. Specifically, this study introduces a new component into the system in order to assess a complete scenario about angiogenesis. This is important because oxygen is the main nutrient for tumor spread. The extended model presented in this paper is supported by a theoretical framework. Hence, a scheme for finding a numerical solution of the model is proposed. Then, through analytical and numerical analysis, we explore the implications of this extension. Numerical experiments show the reliability of the proposed scheme by preserving the properties of continuous counterpart.

The rest of paper is organized as follows: in Section 2 introduces the extended analytical PDEs model, accompanied by its analytical properties. Section 3 illustrates the proposed numerical scheme supported by theoretical foundations of the model. In Section 4 exhibits the algorithm related to scheme and numerical experiments for confirming theoretical results. Finally, Section 5 closes the paper with conclusions.

## Mathematical model and analytical properties

In this section we propose a mathematical model for tumor angiogenesis with oxygen dynamics extends a reaction-diffusion framework studied in De Luca and Marcellino (2025). We consider a one-dimensional spatial domain $\Omega = [0, L_f]$ with $x \in \Omega$ representing the spatial variable and $t \in [0, \infty )$ denoting time. The model describes the spatiotemporal evolution of five key variables: endothelial cell density *C*(*x*, *t*), protease concentration *P*(*x*, *t*), inhibitor concentration *I*(*x*, *t*), extracellular matrix density *F*(*x*, *t*), and oxygen concentration *O*(*x*, *t*). The governing equations constitute a system of nonlinear PDEs:

1 $$\begin{aligned} \frac{\partial C}{\partial t}&= \frac{\partial }{\partial x}\left[ d_c\frac{\partial C}{\partial x} - C\left( \alpha _1\frac{\partial F}{\partial x} - \alpha _2\frac{\partial I}{\partial x} + \alpha _3\frac{\partial \phi }{\partial x}\right) \right] + k_1C(1-C)H(O), \end{aligned}$$

2 $$\begin{aligned} \frac{\partial P}{\partial t}&= d_P\frac{\partial ^2 P}{\partial x^2} - k_3PI + k_4TC + k_5T - k_6P, \end{aligned}$$

3 $$\begin{aligned} \frac{\partial I}{\partial t}&= d_I\frac{\partial ^2 I}{\partial x^2} - k_3PI, \end{aligned}$$

4 $$\begin{aligned} \frac{\partial F}{\partial t}&= -k_2PF, \end{aligned}$$

5 $$\begin{aligned} \frac{\partial O}{\partial t}&= d_O\frac{\partial ^2 O}{\partial x^2} - \frac{\gamma _1 CO}{K_O + O} + \gamma _2(O_{max} - O)\beta C, \quad x \in \Omega , t > 0. \end{aligned}$$

The endothelial cell density equation includes diffusion (with coefficient $d_c > 0$), chemotaxis and haptotaxis (with sensitivity coefficients $\alpha _2 < 0$, $\alpha _1 > 0$, and $\alpha _3 > 0$), and oxygen-dependent proliferation through the Michaelis-Menten function (Srinivasan 2022)

$$\begin{aligned} H(O) = \frac{O}{K_O + O}. \end{aligned}$$

The protease, inhibitor, and extracellular matrix equations capture the interactions between these components, while the oxygen equation models the balance between diffusion, consumption by cells, and supply from blood vessels. The oxygen concentration *O*(*x*, *t*) is governed by a reaction-diffusion equation incorporating diffusion (with coefficient $d_O > 0$), consumption by endothelial cells (with maximum rate $\gamma _1 > 0$ and half-saturation constant $K_O > 0$), and supply from blood vessels (with rate parameter $\gamma _2 > 0$, vessel functionality coefficient $\beta > 0$, and maximum oxygen concentration $O_{max}$). The consumption term $\frac{\gamma _1 CO}{K_O + O}$ employs Michaelis-Menten kinetics to capture the saturation of oxygen consumption at high oxygen concentrations (Cornish-Bowden 2015). The supply term $\gamma _2(O_{max} - O)\beta C$ models oxygen delivery from functional blood vessels, with the rate proportional to both the density of vessels and the difference between the current oxygen concentration and the maximum possible concentration $O_{max}$.

The tumor angiogenic factor distribution is represented by

$$\begin{aligned} T(x) = \exp \left( -\epsilon ^{-1}(L_f - x)^2\right) , \end{aligned}$$

where $\epsilon > 0$ controls the width of the transition zone between tumor and normal tissue. Since *T*(*x*) depends only from the space, a regularized version

$$\begin{aligned} \phi (x) = \alpha _4^{-1}\log (1 + \alpha _4 T(x)) \end{aligned}$$

is introduced to avoid numerical instabilities (De Luca et al. 2025). The system is subject to (Neumann) no-flux boundary conditions:

6 $$\begin{aligned} \frac{\partial u}{\partial x}(0, t) = \frac{\partial u}{\partial x}(L_f, t) = 0, \quad \text {for } u = C, P, I, F, O. \end{aligned}$$

The initial conditions are specified as:

7 $$\begin{aligned} C(x, 0)&= {\left\{ \begin{array}{ll} C_0, & 0 \le x \le a, \\ 0, & a < x \le L_f, \end{array}\right. } \end{aligned}$$

8 $$\begin{aligned} P(x, 0)&= \xi _1, \quad I(x, 0) = \xi _2, \quad F(x, 0) = \xi _3, \end{aligned}$$

9 $$\begin{aligned} O(x, 0)&= O_0e^{-\lambda x} + \xi _4, \end{aligned}$$

where $\xi _i$ ($i = 1, 2, 3, 4$) are small positive constants. The exponential profile for oxygen reflects the diffusion-limited oxygen distribution from the parent vessel.

Let us first rewrite the system in a more compact form. Define the vector $\textbf{u} = (C, P, I, F, O)^T$, where *C*, *P*, *I*, *F*, and *O* represent the endothelial cell density, protease concentration, inhibitor concentration, extracellular matrix density, and oxygen concentration, respectively. The system (1) can be expressed as:

10 $$\begin{aligned} \frac{\partial \textbf{u}}{\partial t} = \mathcal {A}(\textbf{u}) + \mathcal {F}(\textbf{u}), \end{aligned}$$

where $\mathcal {A}(\textbf{u})$ represents the differential operator that captures diffusion, chemotaxis, and haptotaxis effects, while $\mathcal {F}(\textbf{u})$ includes the reaction terms. Specifically,

11 $$\begin{aligned} \mathcal {A}(\textbf{u}) = \begin{pmatrix} \frac{\partial }{\partial x}\left[ d_c\frac{\partial C}{\partial x} - C\left( \alpha _1\frac{\partial F}{\partial x} - \alpha _2\frac{\partial I}{\partial x} + \alpha _3\frac{\partial \varphi }{\partial x}\right) \right] \\ d_P\frac{\partial ^2 P}{\partial x^2} \\ d_I\frac{\partial ^2 I}{\partial x^2} \\ 0 \\ d_O\frac{\partial ^2 O}{\partial x^2} \end{pmatrix}, \end{aligned}$$

and

12 $$\begin{aligned} \mathcal {F}(\textbf{u}) = \begin{pmatrix} k_1C(1-C)H(O) \\ -k_3PI + k_4TC + k_5T - k_6P \\ -k_3PI \\ -k_2PF \\ -\frac{\gamma _1CO}{K_O + O} + \gamma _2(O_{max} - O)\beta C \end{pmatrix}. \end{aligned}$$

We analyze the system in the function space $X = [L^2(\Omega )]^5$, with the standard $L^2$ norm, and define the domain

$$\begin{aligned} D(\mathcal {A}) = \{\textbf{u} \in [H^2(\Omega )]^5 : \frac{\partial \textbf{u}}{\partial x}(0, t) = \frac{\partial \textbf{u}}{\partial x}(L_f, t) = 0\}. \end{aligned}$$

We establish the existence and uniqueness of solutions using semigroup theory combined with the method of upper and lower solutions for reaction-diffusion systems with cross-diffusion terms (Hernandez-Aristizabal et al. 2021).

### Theorem 1

(Existence and Uniqueness). Let $\Omega = [0, L_f]$ and $T > 0$. Assume that:

1. The initial data $\textbf{u}_0 = (C_0, \xi _1, \xi _2, \xi _3, O_0e^{-\lambda x} + \xi _4)$ satisfies $\textbf{u}_0 \in [L^\infty (\Omega )]^5$ with $C_0 \in [0, 1]$, $\xi _i > 0$ for $i = 1, 2, 3, 4$, and $O_0 > 0$.
2. The coefficients satisfy $d_C, d_P, d_I, d_O > 0$, $\alpha _1, \alpha _3 > 0$, $\alpha _2 < 0$, $k_i > 0$ for $i = 1, \ldots , 6$, $\gamma _1, \gamma _2, K_O, O_{max}, \beta > 0$.
3. $$\varphi \in C^2(\Omega )$$ and $$T \in C(\Omega )$$ with $$0 \le T \le 1$$.

Then there exists a unique solution $\textbf{u} \in C([0, T]; [L^2(\Omega )]^5) \cap L^2(0, T; [H^1(\Omega )]^5)$ to the system (1) with zero-flux boundary conditions (6) and initial conditions (7).

### Proof

We divide the proof into several steps:

**Step 1:** For the linear part, we show that the operator $\mathcal {A}$ generates a $C_0$-semigroup of contractions on *X*. First, we observe that the pure diffusion terms in $\mathcal {A}$ generate analytic semigroups. Let $\mathcal {A}_0$ be the operator containing only the diffusion terms:
  13 $$\begin{aligned} \mathcal {A}_0(u) = \begin{pmatrix} d_C\frac{\partial ^2 C}{\partial x^2} \\ d_P\frac{\partial ^2 P}{\partial x^2} \\ d_I\frac{\partial ^2 I}{\partial x^2} \\ 0 \\ d_O\frac{\partial ^2 O}{\partial x^2} \end{pmatrix}. \end{aligned}$$
  It is well-established that $\mathcal {A}_0$ with domain $D(\mathcal {A}_0) = D(\mathcal {A})$ generates an analytic semigroup on *X*. To prove that $\mathcal {A}$ generates a $C_0$-semigroup, we decompose $\mathcal {A} = \mathcal {A}_0 + \mathcal {A}_1$ where $\mathcal {A}_0$ contains the diffusion terms and $\mathcal {A}_1$ contains the advection-type terms. The remaining terms in $\mathcal {A}_1$ involve first-order spatial derivatives of *C*, *I*, *F*, and $\phi$. Since $C \in H^1(\Omega )$, $I, F \in H^2(\Omega )$ and $\phi \in C^2(\Omega )$, we have that $\frac{\partial C}{\partial x} \in L^2(\Omega )$, $\frac{\partial I}{\partial x}, \frac{\partial F}{\partial x}, \frac{\partial \phi }{\partial x} \in H^1(\Omega ) \hookrightarrow C(\Omega )$. Furthermore, assuming $C \in L^{\infty }(\Omega )$, these terms represent relatively bounded perturbations of $\mathcal {A}_0$. For any $u \in D(\mathcal {A}_0)$, we estimate
  14 $$\begin{aligned} \Vert \mathcal {A}_1 u\Vert _X \le M_1\Vert u\Vert _{H^1(\Omega )^5} \le M_2(\Vert \mathcal {A}_0 u\Vert _X + \Vert u\Vert _X) \end{aligned}$$
  with constants $M_1, M_2 > 0$ depending on $\alpha _1, \alpha _2, \alpha _3$ and the maximum values of *C*, *I*, *F*, and $\phi$. By the perturbation theorem for semigroup generators (Goldstein 1974), $\mathcal {A}$ generates a $C_0$-semigroup on *X*.
**Step 2:** We establish local Lipschitz continuity of the reaction terms $\mathcal {F}$. For any $u = (C, P, I, F, O)^T$ and $v = (C', P', I', F', O')^T$ in a bounded subset of $[L^2(\Omega )]^5$, we have:
  15 $$\begin{aligned} \Vert \mathcal {F}(u) - \mathcal {F}(v)\Vert _X \le L(u, v)\Vert u - v\Vert _X, \end{aligned}$$
  where *L*(*u*, *v*) depends continuously on $\Vert u\Vert _X$ and $\Vert v\Vert _X$. This follows from the specific form of $\mathcal {F}$ and the properties of the Michaelis-Menten function $H(O) = \frac{O}{K_O+O}$, which is bounded and Lipschitz continuous for $O \ge 0$.
**Step 3:** By the standard theory of semilinear evolution equations (Henry 1981), the local Lipschitz property of $\mathcal {F}$ combined with the generation of a $C_0$-semigroup by $\mathcal {A}$ ensures the local existence of a unique mild solution $\textbf{u} \in C([0, T_{max}); X)$ for some maximal time of existence $T_{max} > 0$.
**Step 4:** To establish global existence, we need to derive a priori bounds to ensure that the solution cannot blow up in finite time. As demonstrated in Theorem 2, the components of the solution satisfy the following uniform bounds for all $(x,t) \in \Omega \times [0,\infty )$:
  13 $$\begin{aligned} 0 \le C(x,t)&\le 1, \end{aligned}$$
  14 $$\begin{aligned} 0 \le P(x,t)&\le \max \left\{ \xi _1, \frac{k_4T_{\max } + k_5T_{\max }}{k_6}\right\} , \end{aligned}$$
  15 $$\begin{aligned} 0 \le I(x,t)&\le \xi _2, \end{aligned}$$
  16 $$\begin{aligned} 0 \le F(x,t)&\le \xi _3, \end{aligned}$$
  17 $$\begin{aligned} 0 \le O(x,t)&\le \max \{O_0 + \xi _4, O_{\max }\}, \end{aligned}$$
  where $T_{\max } = \max _{x \in \Omega } T(x) \le 1$. These uniform bounds ensure that the solution remains in a bounded subset of
  $$\begin{aligned} [L^\infty (\Omega )]^5\quad \forall t \in [0,T_{\max }). \end{aligned}$$
  This prevents blow-up in finite time, and therefore $T_{\max } = \infty$, establishing the global existence of solutions.
**Step 5:** The regularity of the solution follows from semigroup theory and the smoothing properties of the diffusion terms. By standard parabolic regularity theory (Ladyzhenskaya et al. 1968), if $\textbf{u}_0 \in [L^2(\Omega )]^5$, then $\textbf{u} \in C((0, T]; [H^2(\Omega )]^5)$ for any $T > 0$. $\square$

### Theorem 2

(A priori estimates.) Let the assumptions of Theorem 1 hold. Then any solution $u = (C, P, I, F, O)^T$ to the system (1) satisfies the following estimates $\forall (x,t) \in \Omega \times [0,\infty )$:

18 $$\begin{aligned} 0 \le C(x,t)&\le 1, \end{aligned}$$

19 $$\begin{aligned} 0 \le P(x,t)&\le \max \left\{ \xi _1, \frac{k_4T_{max} + k_5T_{max}}{k_6}\right\} , \end{aligned}$$

20 $$\begin{aligned} 0 \le I(x,t)&\le \xi _2, \end{aligned}$$

21 $$\begin{aligned} 0 \le F(x,t)&\le \xi _3, \end{aligned}$$

22 $$\begin{aligned} 0 \le O(x,t)&\le \max \{O_0 + \xi _4, O_{max}\}, \end{aligned}$$

where $$T_{max} = \max _{x \in \Omega } T(x) \le 1$$.

### Proof

Taking into account the endothelial cell density, the maximum principle for parabolic equations applied to first equation in (1) yields $0 \le C(x,t) \le 1.$ About proteases concentration, when *P*(*x*, *t*) is greater than $\frac{k_4T_{max} + k_5T_{max}}{k_6}$, the term $-k_6$ dominates in the second equation of (1) by ensuring $\frac{\partial P}{\partial t} < 0$. The non-negativity of *I* and *F* follows from their initial conditions and the structure of equations (7), while their upper bounds are preserved due to the non-positive reaction terms. Regarding to the oxygen concentration, implies that when *O* approaches its upper bound, the consumption term dominates the supply term, establishing the result. $\square$

We now establish an important result regarding the structural stability of the system, which is crucial for understanding the long-term behavior of solutions and the robustness of numerical approximations.

### Theorem 3

(Structural Stability and Dissipativity) Under the assumptions of Theorem 1, the system (1) is equipped by a global attractor in $X = [L^2(\Omega )]^5$. Moreover, the system exhibits the following dissipative property: there exists a bounded absorbing set $\mathcal {B} \subset X$ such that for any bounded set $B \subset X$ of initial data, there exists a time $T_B > 0$ such that $\textbf{u}(t) \in \mathcal {B}$ for all $t \ge T_B$ and all initial data $\textbf{u}_0 \in B$.

### Proof

To establish the existence of a global attractor, we need to verify that the semigroup $\{S(t)\}_{t\ge 0}$ generated by the system is dissipative and asymptotically compact (Temam 1997).

**Step 1:** We first define an appropriate energy functional:
  23 $$\begin{aligned} E(t) = \frac{1}{2}\int _{\Omega }\left( C^2 + P^2 + I^2 + F^2 + \frac{1}{\gamma _2\beta }O^2\right) \,dx. \end{aligned}$$
  Differentiating with respect to time and using equations (1)–(5), and applying integration by parts with the zero-flux boundary conditions, we obtain:
  24 $$\begin{aligned} \frac{dE}{dt} + \int _{\Omega }\left( d_C|\nabla C|^2 + d_P|\nabla P|^2 + d_I|\nabla I|^2 + \frac{d_O}{\gamma _2\beta }|\nabla O|^2\right) \,dx = \int _{\Omega }R\,dx, \end{aligned}$$
  where *R* represents the contributions from the reaction terms:
  25 $$\begin{aligned} R&= C \cdot k_1C(1-C)H(O) + P \cdot (-k_3PI + k_4TC + k_5T - k_6P) \nonumber \\&\quad + I \cdot (-k_3PI) + F \cdot (-k_2PF) + \frac{O}{\gamma _2\beta } \cdot \left( - \frac{\gamma _1CO}{K_O + O} + \gamma _2(O_{max} - O)\beta C\right) . \end{aligned}$$
**Step 2:** Using the a priori bounds established in Theorem 2 and the specific form of the reaction terms, we can derive an inequality of the form:
  26 $$\begin{aligned} \frac{dE}{dt} + \mu E \le K, \end{aligned}$$
  for some constants $\mu > 0$ and $K > 0$, which implies:
  27 $$\begin{aligned} E(t) \le E(0)e^{-\mu t} + \frac{K}{\mu }(1 - e^{-\mu t}). \end{aligned}$$
  This shows that the energy functional is eventually bounded by $\frac{K}{\mu }$, regardless of the initial data, establishing the dissipativity of the system.
**Step 3:** To show asymptotic compactness, we use the fact that the diffusion terms provide a smoothing effect. For any sequence $\{t_n\}$ with $t_n \rightarrow \infty$ and any bounded sequence of initial data $\{\textbf{u}_{0,n}\}$, the sequence $\{S(t_n)\textbf{u}_{0,n}\}$ is relatively compact in *X*. This follows from the a priori estimates and the compactness of the embedding
  $$\begin{aligned} H^1(\Omega ) \hookrightarrow L^2(\Omega ). \end{aligned}$$
  Using the variation of constants formula and the smoothing properties of the semigroup, we can show that for sufficiently large *t*, $\textbf{u}(t)$ belongs to a compact subset of *X* that depends only on the bounds of the initial data (Henry 1981).
**Step 4:** By the standard theory of infinite-dimensional dynamical systems (Temam 1997), the dissipativity and asymptotic compactness of the semigroup ensure the existence of a global attractor $\mathcal {A} \subset X$, which is the smallest compact invariant set that attracts all bounded sets of initial data. The absorbing set $\mathcal {B}$ can be explicitly constructed as:
  28 $$\begin{aligned} \mathcal {B} = \left\{ \textbf{u} \in X : \Vert \textbf{u}\Vert ^2_X \le \frac{2K}{\mu } + 1\right\} , \end{aligned}$$
  which completes the proof. $\square$

## Numerical approach

In order to numerically solve the system of equations (10)–(12), we employ the method of lines approach. This system is then integrated using an appropriate time-stepping method. We present a formulation of this approach, incorporating gradient and Laplacian operators in matrix form and analyzing the stability and accuracy of the resulting numerical scheme.

Let us discretize the spatial domain $\Omega = [0, L_f]$ into M+1 equidistant nodes, creating a uniform grid $\{x_j = j\Delta x\}_{j=0}^M$ where $\Delta x = L_f/M$. We define the corresponding grid function for each variable:

$$\begin{aligned} \begin{array}{cc} C_j(t) \approx C(x_j, t), & P_j(t) \approx P(x_j, t), \\ I_j(t) \approx I(x_j, t), & F_j(t) \approx F(x_j, t), \\ O_j(t) \approx O(x_j, t), & \end{array} \end{aligned}$$

for $j = 0, 1, \ldots , M$. We arrange these values into column vectors:

$$\begin{aligned} \textbf{C}(t) = [C_0(t), C_1(t), \ldots , C_M(t)]^T, \end{aligned}$$

and similarly for $\textbf{P}(t)$, $\textbf{I}(t)$, $\textbf{F}(t)$, and $\textbf{O}(t)$. The spatial discretization employs second-order finite difference approximations with consistent treatment of the Neumann boundary conditions. The discrete gradient operator $\textbf{D}_x \in \mathbb {R}^{(M+1) \times (M+1)}$ is constructed as:

29 $$\begin{aligned} \textbf{D}_x = \frac{1}{2\Delta x} \begin{pmatrix} -3 & 4 & -1 & 0 & \cdots & 0 & 0 \\ -1 & 0 & 1 & 0 & \cdots & 0 & 0 \\ 0 & -1 & 0 & 1 & \cdots & 0 & 0 \\ \vdots & \vdots & \vdots & \vdots & \ddots & \vdots & \vdots \\ 0 & 0 & 0 & -1 & \cdots & 0 & 1 \\ 0 & 0 & 0 & 1 & \cdots & -4 & 3 \end{pmatrix}. \end{aligned}$$

The discrete Laplacian operator $\textbf{D}_{xx} \in \mathbb {R}^{(M+1) \times (M+1)}$ incorporates the homogeneous Neumann conditions:

30 $$\begin{aligned} \textbf{D}_{xx} = \frac{1}{(\Delta x)^2} \begin{pmatrix} -2 & 2 & 0 & 0 & \cdots & 0 & 0 \\ 1 & -2 & 1 & 0 & \cdots & 0 & 0 \\ 0 & 1 & -2 & 1 & \cdots & 0 & 0 \\ \vdots & \vdots & \vdots & \vdots & \ddots & \vdots & \vdots \\ 0 & 0 & 0 & 1 & \cdots & -2 & 1 \\ 0 & 0 & 0 & 0 & \cdots & 2 & -2 \end{pmatrix}. \end{aligned}$$

The boundary rows implement second-order one-sided finite difference stencils: $\textbf{D}_x[0,:] = \frac{1}{2\Delta x}[-3, 4, -1, 0, \ldots ]$ and $\textbf{D}_x[M,:] = \frac{1}{2\Delta x}[\ldots , 0, 1, -4, 3]$, ensuring $\mathcal {O}((\Delta x)^2)$ global truncation error while enforcing $\partial u/\partial x|_{\partial \Omega } = 0$. The discretized system can now be expressed as:

31 $$\begin{aligned} \frac{d\textbf{C}}{dt}&= d_c\,\textbf{D}_{xx}\textbf{C} - \textbf{D}_x\!\big (\textbf{C}\odot (\alpha _1 \textbf{D}_x\textbf{F}-\alpha _2 \textbf{D}_x\textbf{I}+\alpha _3 \textbf{D}_x\boldsymbol{\phi })\big ) + k_1\,\textbf{C}\odot (\textbf{1}-\textbf{C})\odot \textbf{H}(\textbf{O}), \nonumber \\ \frac{d\textbf{P}}{dt}&= d_P\,\textbf{D}_{xx}\textbf{P} - k_3\,\textbf{P}\odot \textbf{I} + k_4\,\textbf{T}\odot \textbf{C} + k_5\,\textbf{T} - k_6\,\textbf{P}, \nonumber \\ \frac{d\textbf{I}}{dt}&= d_I\,\textbf{D}_{xx}\textbf{I} - k_3\,\textbf{P}\odot \textbf{I}, \nonumber \\ \frac{d\textbf{F}}{dt}&= -\,k_2\,\textbf{P}\odot \textbf{F}, \\ \frac{d\textbf{O}}{dt}&= d_O\,\textbf{D}_{xx}\textbf{O} - \frac{\gamma _1\,\textbf{C}\odot \textbf{O}}{K_O\,\textbf{1} + \textbf{O}} + \gamma _2\,(O_{\max }\textbf{1} - \textbf{O})\odot \beta \,\textbf{C},\nonumber \end{aligned}$$

where $\odot$ denotes the Hadamard product, $\textbf{1}$ is a vector of ones, $\boldsymbol{\phi }$ is the discretization of $\phi (x)$, and $\textbf{T}$ is the discretization of the tumor angiogenic factor distribution *T*(*x*). The function $\textbf{H}(\textbf{O})$ is applied element-wise:

$$\begin{aligned} \textbf{H}(\textbf{O})_j = \frac{O_j}{K_O + O_j}. \end{aligned}$$

The resulting semi-discrete system can be written in compact form as:

32 $$\begin{aligned} \frac{d\textbf{U}}{dt} = \textbf{L}(\textbf{U}) + \textbf{N}(\textbf{U}) \end{aligned}$$

where $\textbf{U} = [\textbf{C}^T, \textbf{P}^T, \textbf{I}^T, \textbf{F}^T, \textbf{O}^T]^T$ is the state vector, $\textbf{L}(\textbf{U})$ contains the linear diffusion terms, and $\textbf{N}(\textbf{U})$ contains the nonlinear advection and reaction terms:

$$\textbf{L}(\textbf{U}) = \begin{bmatrix} d_c \textbf{D}_{xx}\textbf{C} \\ d_P \textbf{D}_{xx}\textbf{P} \\ d_I \textbf{D}_{xx}\textbf{I} \\ \textbf{0} \\ d_O \textbf{D}_{xx}\textbf{O} \end{bmatrix},$$

$$\textbf{N}(\textbf{U}) = \begin{bmatrix} -\textbf{D}_x(\textbf{C} \odot (\alpha _1 \textbf{D}_x\textbf{F} - \alpha _2 \textbf{D}_x\textbf{I} + \alpha _3 \textbf{D}_x\boldsymbol{\phi })) + k_1 \textbf{C} \odot (\textbf{1} - \textbf{C}) \odot \textbf{H}(\textbf{O}) \\ -k_3 \textbf{P} \odot \textbf{I} + k_4 \textbf{T} \odot \textbf{C} + k_5 \textbf{T} - k_6 \textbf{P} \\ -k_3 \textbf{P} \odot \textbf{I} \\ -k_2 \textbf{P} \odot \textbf{F} \\ -\frac{\gamma _1 \textbf{C} \odot \textbf{O}}{K_O \textbf{1} + \textbf{O}} + \gamma _2 (O_{max}\textbf{1} - \textbf{O}) \odot \beta \textbf{C} \end{bmatrix}.$$

A particular consideration in the numerical solution of this system is the stiffness induced by the diffusion terms, which impose strict stability restrictions on explicit time-stepping methods. The classical stability analysis yields the condition $\Delta t \le \frac{(\Delta x)^2}{2d_{max}}$ where $d_{max} = \max \{d_c, d_P, d_I, d_O\}$. This condition can be prohibitively restrictive for fine spatial grids or large diffusion coefficients.

### Theorem 4

(Stability Constraints for Explicit Integration). Let $\textbf{U} = (\textbf{C}, \textbf{P}, \textbf{I}, \textbf{F}, \textbf{O})^T \in \mathbb {R}^{5(M+1)}$ denote the discretized state vector of system (31). Consider the semi-discrete system:

$$\begin{aligned} \frac{d\textbf{U}}{dt} = \textbf{L}(\textbf{U}) + \textbf{N}(\textbf{U}) \end{aligned}$$

where

$$\begin{aligned} \textbf{L}: \mathbb {R}^{5(M+1)} \rightarrow \mathbb {R}^{5(M+1)}\quad \text {and}\quad \textbf{N}: \mathbb {R}^{5(M+1)} \rightarrow \mathbb {R}^{5(M+1)} \end{aligned}$$

represents the linear differential operators and the nonlinear reaction terms, respectively.

Set the step size $\Delta t > 0$, the stability for explicit time integration requires:

1. **Diffusion constraint**: $\Delta t \le \frac{(\Delta x)^2}{2d_{\max }}$ where $d_{\max } = \max \{d_C, d_P, d_I, d_O\}.$
2. **Advection constraint**: $\Delta t \le C_{CFL} \Delta x$ for some constant $C_{CFL} > 0$ depending on $\{\alpha _1, \alpha _2, \alpha _3\}.$

The overall stability condition is $\Delta t \le \min \left\{ \frac{(\Delta x)^2}{2d_{\max }}, C_{CFL}\Delta x\right\} = \mathcal {O}((\Delta x)^2)$.

### Proof

We employ von Neumann stability analysis under the frozen coefficient assumption. Let $\mathcal {U} \subset \mathbb {R}^{5(M+1)}$ be a bounded invariant region for system (32). For $\textbf{U} \in \mathcal {U}$, consider the linearization:

$$\begin{aligned} \frac{d\textbf{V}}{dt} = \textbf{A}(\textbf{U})\textbf{V} \end{aligned}$$

where $\textbf{A}(\textbf{U}) = \textbf{J}_L(\textbf{U}) + \textbf{J}_N(\textbf{U})$ with $\textbf{J}_L$, $\textbf{J}_N$ denoting the Jacobians of $\textbf{L}$ and $\textbf{N}$ respectively. For the Fourier mode $\textbf{v}_j^{(k)} = \hat{\textbf{v}}^{(k)} e^{i\xi j\Delta x}$ where $k \in \{C,P,I,F,O\}$ and $\xi \in [0, 2\pi /\Delta x]$, the spectral radius condition for stability is:

$$\begin{aligned} \rho (\textbf{I} + \Delta t \hat{\textbf{A}}(\xi )) \le 1 + C\Delta t \end{aligned}$$

for some constant $C > 0$ and all admissible $\xi$.

**Step 1**: (Diffusion terms): The eigenvalues of the discretized Laplacian $\textbf{D}_{xx}$ are:
  $$\begin{aligned} \lambda _m = -\frac{4}{\Delta x^2}\sin ^2\left( \frac{m\pi \Delta x}{2L_f}\right) , \quad m = 0,1,\ldots ,M. \end{aligned}$$
  The amplification factor for explicit Euler applied to $d_{\max }\textbf{D}_{xx}$ gives:
  $$\begin{aligned} |1 + \Delta t \lambda _m| \le 1 \iff \Delta t \le \frac{(\Delta x)^2}{2d_{\max }}. \end{aligned}$$
**Step 2**: (Advection terms): For the chemotactic/haptotactic operators in $\textbf{N}$, the CFL condition emerges from the hyperbolic character of the advective transport, yielding $\Delta t \le C_{CFL}\Delta x$. The assertion follows from the spectral bound $\max \{2d_{\max }/(\Delta x)^2, C_{CFL}/\Delta x\}$. $\square$

### Remark

Although we employ a fully explicit RK4 integration treating $\textbf{L}(\textbf{U})$ and $\textbf{N}(\textbf{U})$ simultaneously, the decomposition facilitates stability analysis and provides a natural framework for future extensions to operator splitting methods (such as IMEX schemes) when dealing with stiffer parameter regimes. The stability analysis above establishes the fundamental constraints for explicit integration of the decomposed system and provides the theoretical foundation for both current implementation and potential future modifications. Meanwhile, for the specific parameter regimes of interest in tumor angiogenesis, the stability restriction is manageable with moderate grid sizes. The fully discrete scheme proceeds as follows. Given the approximation $\textbf{U}^n$ at time $t_n$, we compute $\textbf{U}^{n+1}$ at time $t_{n+1} = t_n + \Delta t$ using:

33 $$\begin{aligned} \begin{aligned} \textbf{k}_1&= \textbf{L}(\textbf{U}^n) + \textbf{N}(\textbf{U}^n), \\ \textbf{k}_2&= \textbf{L}(\textbf{U}^n + \frac{\Delta t}{2}\textbf{k}_1) + \textbf{N}(\textbf{U}^n + \frac{\Delta t}{2}\textbf{k}_1), \\ \textbf{k}_3&= \textbf{L}(\textbf{U}^n + \frac{\Delta t}{2}\textbf{k}_2) + \textbf{N}(\textbf{U}^n + \frac{\Delta t}{2}\textbf{k}_2), \\ \textbf{k}_4&= \textbf{L}(\textbf{U}^n + \Delta t\textbf{k}_3) + \textbf{N}(\textbf{U}^n + \Delta t\textbf{k}_3), \\ \textbf{U}^{n+1}&= \textbf{U}^n + \frac{\Delta t}{6}(\textbf{k}_1 + 2\textbf{k}_2 + 2\textbf{k}_3 + \textbf{k}_4). \end{aligned} \end{aligned}$$

We now establish the convergence properties of this numerical scheme:

### Theorem 5

(Convergence of the Numerical Scheme). Let

$$\begin{aligned} \textbf{u}(t) \in C^1([0,T]; \mathcal {X}) \cap C([0,T]; \mathcal {Y}) \end{aligned}$$

be the unique solution to system (10) with $\mathcal {X} = [L^2(\Omega )]^5$ and $\mathcal {Y} = [H^2(\Omega )]^5$. Let $\{\textbf{U}^n\}_{n=0}^N$ be the numerical approximation obtained by the method of lines with central difference spatial discretization and RK4 temporal integration, where $t_n = n\Delta t$ and $N\Delta t = T$.

Under the stability constraint $\Delta t \le C_0(\Delta x)^2$ for sufficiently small $C_0 > 0$, there exist constants $K_1, K_2 > 0$ such that:

$$\begin{aligned} \max _{0 \le n \le N} \Vert \textbf{U}^n - \textbf{u}(t_n)\Vert _{\mathcal {X}} \le K_1(\Delta x)^2 + K_2(\Delta t)^4. \end{aligned}$$

### Proof

The convergence follows from the Lax Equivalence Theorem by establishing consistency and stability. The spatial discretization via central differences introduces a local truncation error of order $\mathcal {O}((\Delta x)^2)$ for sufficiently smooth solutions, while the RK4 temporal integration contributes $\mathcal {O}((\Delta t)^5)$ local error per step. About stability, we analyze the discrete evolution operator $S_{\Delta t}: \mathcal {X}_h \rightarrow \mathcal {X}_h$ on the finite-dimensional subspace $\mathcal {X}_h \subset \mathbb {R}^{5(M+1)}$. The nonlinear operator $\textbf{F}(\textbf{U}) = \textbf{L}(\textbf{U}) + \textbf{N}(\textbf{U})$ satisfies the Lipschitz condition

$$\begin{aligned} \Vert \textbf{F}(\textbf{V}) - \textbf{F}(\textbf{W})\Vert _{\mathcal {X}} \le L\Vert \textbf{V} - \textbf{W}\Vert _{\mathcal {X}} \end{aligned}$$

for $\textbf{V}, \textbf{W}$ in the bounded invariant region $\mathcal {U}$ established by Theorem 2. This Lipschitz structure is preserved by the RK4 scheme under the stability constraint, yielding

$$\begin{aligned} \Vert S_{\Delta t}(\textbf{V}) - S_{\Delta t}(\textbf{W})\Vert _{\mathcal {X}} \le (1 + L\Delta t)\Vert \textbf{V} - \textbf{W}\Vert _{\mathcal {X}}. \end{aligned}$$

The global discretization error $\textbf{e}^n = \textbf{U}^n - \textbf{u}(t_n)$ satisfies the recurrence

$$\begin{aligned} \textbf{e}^{n+1} = S_{\Delta t}(\textbf{u}(t_n) + \textbf{e}^n) - \textbf{u}(t_{n+1}), \end{aligned}$$

which decomposes as the sum of the propagated previous error and the local truncation error:

$$\begin{aligned} \Vert \textbf{e}^{n+1}\Vert \le (1 + L\Delta t)\Vert \textbf{e}^n\Vert + C_{\tau }[(\Delta x)^2 + (\Delta t)^4] \end{aligned}$$

for some constant $C_{\tau }$. Applying the discrete Grönwall inequality with exact initial data $\textbf{e}^0 = \textbf{0}$ yields

$$\begin{aligned} \Vert \textbf{e}^n\Vert \le \frac{C_{\tau }T e^{LT}}{L}[(\Delta x)^2 + (\Delta t)^4], \end{aligned}$$

establishing the result with $K_1 = K_2 = \frac{C_{\tau }T e^{LT}}{L}$.$\square$

### Remark

The Lipschitz constant *L* can be computed explicitly from the bounds established in Theorem 2 and the parameter values of the model.

An essential aspect of numerical schemes for reaction-diffusion-advection systems in biological applications is the preservation of positivity and boundedness of the solution components. The following theorem addresses these properties:

### Theorem 6

(Positivity and Invariant Region Preservation) Define the invariant region

$$\begin{aligned} \mathcal {R} = [0,1]^{M+1} \times [0,P_{\max }]^{M+1} \times [0,\xi _2]^{M+1} \times [0,\xi _3]^{M+1} \times [0,O_{\max }]^{M+1} \end{aligned}$$

where $P_{\max } = \max \{\xi _1, (k_4+k_5)/k_6\}$ and $O_{\max } = \max \{O_0+\xi _4, O_{\max }^*\}$. Under the time step restriction

$$\begin{aligned} \Delta t \le \min \{(\Delta x)^2/(2d_{\max }), 1/L_{\mathcal {R}}, \Delta x/\alpha _{\max }\}, \end{aligned}$$

where $L_{\mathcal {R}}$ is the Lipschitz constant on $\mathcal {R}$ and $\alpha _{\max } = \max \{|\alpha _1|,|\alpha _2|,|\alpha _3|\}$, the RK4 scheme preserves $\mathcal {R}$.

### Proof

The preservation follows from analyzing the sign of the right-hand side at the boundary of each component’s domain. For the endothelial cell density *C*, when $C_j = 0$ the proliferation term $k_1 C_j(1-C_j)H(O_j) = 0$ ensures non-negativity, while at $C_j = 1$ the factor $(1-C_j) = 0$ prevents overflow. Similarly, for the protease concentration *P*, non-negativity at $P_j = 0$ is guaranteed by the production terms $k_4 T_j C_j + k_5 T_j \ge 0$, while the upper bound $P_{\max }$ is preserved since when $P_j \ge (k_4+k_5)/k_6$, the decay term $-k_6 P_j$ dominates the production terms, yielding $dP_j/dt \le 0$.

The inhibitor and matrix components *I*, *F* have no positive source terms, ensuring

$$\begin{aligned} I_j(t) \le I_j(0) = \xi _2\quad \text {and}\quad F_j(t) \le F_j(0) = \xi _3 \quad \forall t \ge 0. \end{aligned}$$

About the oxygen concentration *O*, the supply term $\gamma _2(O_{\max }^* - O_j)\beta C_j$ becomes non-positive when $O_j \ge O_{\max }^*$, while consumption $\gamma _1 C_j O_j/(K_O + O_j) \ge 0$ maintains non-negativity. The convexity of $\mathcal {R}$ and the established boundary behavior ensure that all intermediate stages of RK4 remain within $\mathcal {R}$, completing the preservation property. The explicit time step constants are

$$\begin{aligned} C_1 = 1/(2d_{\max })\quad \text {and} \quad C_2 = \min \{1/\Vert \textbf{J}_{\mathcal {F}}\Vert _{\mathcal {R}}, 1/\alpha _{\max }\} \end{aligned}$$

for diffusion stability and invariant region preservation respectively, where $\textbf{J}_{\mathcal {F}}$ denotes the Jacobian of the nonlinear operator evaluated over $\mathcal {R}$. $\square$

The numerical approach described provides a framework for simulating the complex dynamics of tumor angiogenesis described by proposed model. The method of lines approach combined with RK4 time integration ensures accuracy in both space and time, subject to the necessary stability constraints. The theoretical guarantees established in Theorems 3–5 provide confidence in the reliability of numerical simulations and their fidelity to the underlying mathematical model. While we have chosen a fully explicit approach without operator splitting for simplicity and accuracy, it is worth noting that for practical simulations with very fine spatial grids or in parameter regimes with large diffusion coefficients, alternative approaches such as implicit-explicit methods or adaptive time-stepping strategies may be more efficient. These considerations would be particularly relevant for extending the model to higher spatial dimensions or incorporating additional biological complexity.

## Algorithm and numerical experiments

The previous analyses introduced in previous section lead to the following compact algorithm of the numerical scheme:

**Algorithm 1:**
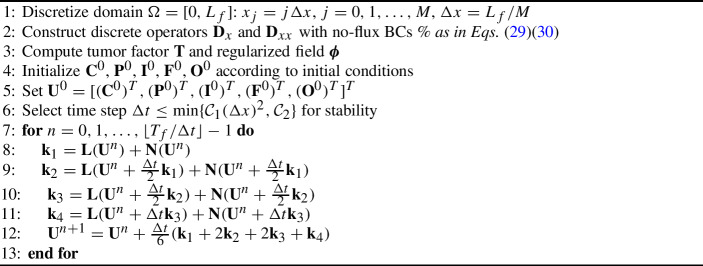
Fully Discrete Scheme for Tumor Angiogenesis Model

We present numerical tests to validate the proposed scheme and demonstrate its properties. More specifically, we run a MATLAB code based on the Algorithm 1. First, we verify the numerical convergence rates through manufactured solutions, then simulate tumor angiogenesis with physiologically relevant parameters, and finally verify the preservation of solution bounds.

**Test 1. Convergence analysis.** To verify the order of convergence of our numerical scheme, we employ the method of manufactured solutions. We consider the following analytical solutions:

34 $$\begin{aligned} C_{\text {exact}}(x,t)&= \frac{1}{2}\left( 1 + \sin (\pi x)\right) e^{-0.1t},\end{aligned}$$

35 $$\begin{aligned} P_{\text {exact}}(x,t)&= 0.2\cos (\pi x)e^{-0.2t},\end{aligned}$$

36 $$\begin{aligned} I_{\text {exact}}(x,t)&= 0.2\sin (2\pi x)e^{-0.3t},\end{aligned}$$

37 $$\begin{aligned} F_{\text {exact}}(x,t)&= 1 - 0.2\sin ^2(\pi x)e^{-0.4t},\end{aligned}$$

38 $$\begin{aligned} O_{\text {exact}}(x,t)&= 0.5 + 0.4\cos (\pi x)e^{-0.1t}. \end{aligned}$$

These solutions are designed to satisfy the boundary conditions and maintain the physical constraints of the model. We introduce appropriate source terms to ensure that these functions satisfy the original system of PDEs. The spatial convergence analysis reveals a critical insight often overlooked in numerical methods. In fact, grid refinement does not guarantee monotonic error reduction Table 1.

**Table 1:** Spatial convergence analysis with varying *M* and stability-constrained time steps

*M*	*C* Component		*P* Component		*I* Component		*O* Component	
	$L^2$ Error	Rate	$L^2$ Error	Rate	$L^2$ Error	Rate	$L^2$ Error	Rate
32	8.74e-3	–	1.23e-2	–	1.05e-2	–	5.62e-3	–
64	2.36e-3	1.89	3.32e-3	1.89	2.84e-3	1.89	1.52e-3	1.88
128	6.54e-4	1.85	9.22e-4	1.85	7.89e-4	1.85	4.23e-4	1.85
**256**	1.74e-4	1.91	2.46e-4	1.91	2.12e-4	1.90	1.13e-4	1.90
512	5.25e-5	1.73	7.89e-5	1.64	6.81e-5	1.64	3.74e-5	1.60
1024	1.85e-5	1.50	2.89e-5	1.45	2.51e-5	1.44	1.47e-5	1.35

The spatial convergence analysis reveals the expected second-order accuracy for moderate grid refinements ($M \le 256$), with rates approaching 1.91 for all variables. However, for finer meshes ($M > 512$), the convergence rates deteriorate to approximately 1.35-1.60, indicating the onset of numerical stiffness that limits the effective accuracy. This behavior is consistent with the theoretical prediction that increasing spatial resolution amplifies the stiffness of the semi-discrete system, requiring progressively smaller time steps that eventually dominate the error behavior.

Now we analyze the convergence rates achieved from time discretization. We selected the spatially most accurate grid $(M=256)$. This choice is not arbitrary but derives from a analysis of numerical performance. Fixing the spatial discretization allows us to isolate the temporal discretization errors while maintaining a stable spatial approximation. We exclude the *F* variable from the convergence analysis because it follows a simple ODE without diffusion terms, making its convergence properties fundamentally different from the other variables. The absence of diffusion in the *F* equation means it is not subject to the same stability constraints as the other variables and exhibits primarily reaction-driven dynamics with different convergence characteristics.

**Table 2:** Time convergence analysis using $M=256$ spatial discretization with $T_f=1$

$\Delta t$	*C* Component		*P* Component		*I* Component		*O* Component	
	$L^2$ Error	Rate	$L^2$ Error	Rate	$L^2$ Error	Rate	$L^2$ Error	Rate
1.0e-3	2.15e-5	–	3.04e-5	–	2.61e-5	–	1.42e-5	–
5.0e-4	6.89e-6	1.64	9.73e-6	1.64	8.35e-6	1.65	4.55e-6	1.64
2.5e-4	2.02e-6	1.77	2.85e-6	1.77	2.45e-6	1.77	1.33e-6	1.77
1.25e-4	5.45e-7	1.89	7.70e-7	1.89	6.61e-7	1.89	3.59e-7	1.89
6.25e-5	1.38e-7	1.98	1.95e-7	1.98	1.67e-7	1.98	9.09e-8	1.98

The time convergence analysis demonstrates rates approaching 2.0 rather than the theoretical fourth-order accuracy of RK4, confirming that the semi-stiff nature of the coupled system reduces the effective temporal order of accuracy. This reduction is typical for reaction-diffusion systems where the diffusion terms impose severe stability constraints on explicit time integration methods, effectively limiting the achievable temporal accuracy regardless of the formal order of the time integrator Table 2.

**Test 2. Angiogenesis simulations.** We employ physiologically relevant parameters as detailed in Table 3, following established ranges from the angiogenesis literature (Mohammad Mirzaei et al. 2024).

**Table 3:** Model parameters with biological interpretation and physiological ranges

Parameter	Biological Meaning	Value	Physiological Range
$d_C$	Endothelial cell random motility	0.001	$10^{-4} - 10^{-2}$
$d_P$, $d_I$	Protease/inhibitor diffusivity	0.01	$10^{-3} - 10^{-1}$
$d_O$	Oxygen diffusion coefficient	0.1	$10^{-2} - 1$
$\alpha _1$	Haptotaxis sensitivity (ECM gradient)	0.1	$0.05 - 0.5$
$\alpha _2$	Chemotaxis sensitivity (inhibitor)	-0.1	$-0.2 - -0.05$
$\alpha _3$	Angiogenic factor sensitivity	0.01	$10^{-3} - 0.1$
$k_1$	Endothelial proliferation rate	0.1	$0.05 - 0.3$
$k_2$	ECM degradation by proteases	0.05	$0.01 - 0.2$
$k_3$	Protease-inhibitor binding rate	0.5	$0.1 - 2.0$
$k_6$	Protease decay rate	0.2	$0.1 - 0.5$
$\gamma _1$	Oxygen consumption rate	0.1	$0.05 - 0.3$
$\gamma _2$	Vascular oxygen supply rate	0.05	$0.01 - 0.2$
$K_O$	Oxygen half-saturation constant	0.2	$0.1 - 0.5$

**Fig. 1:**
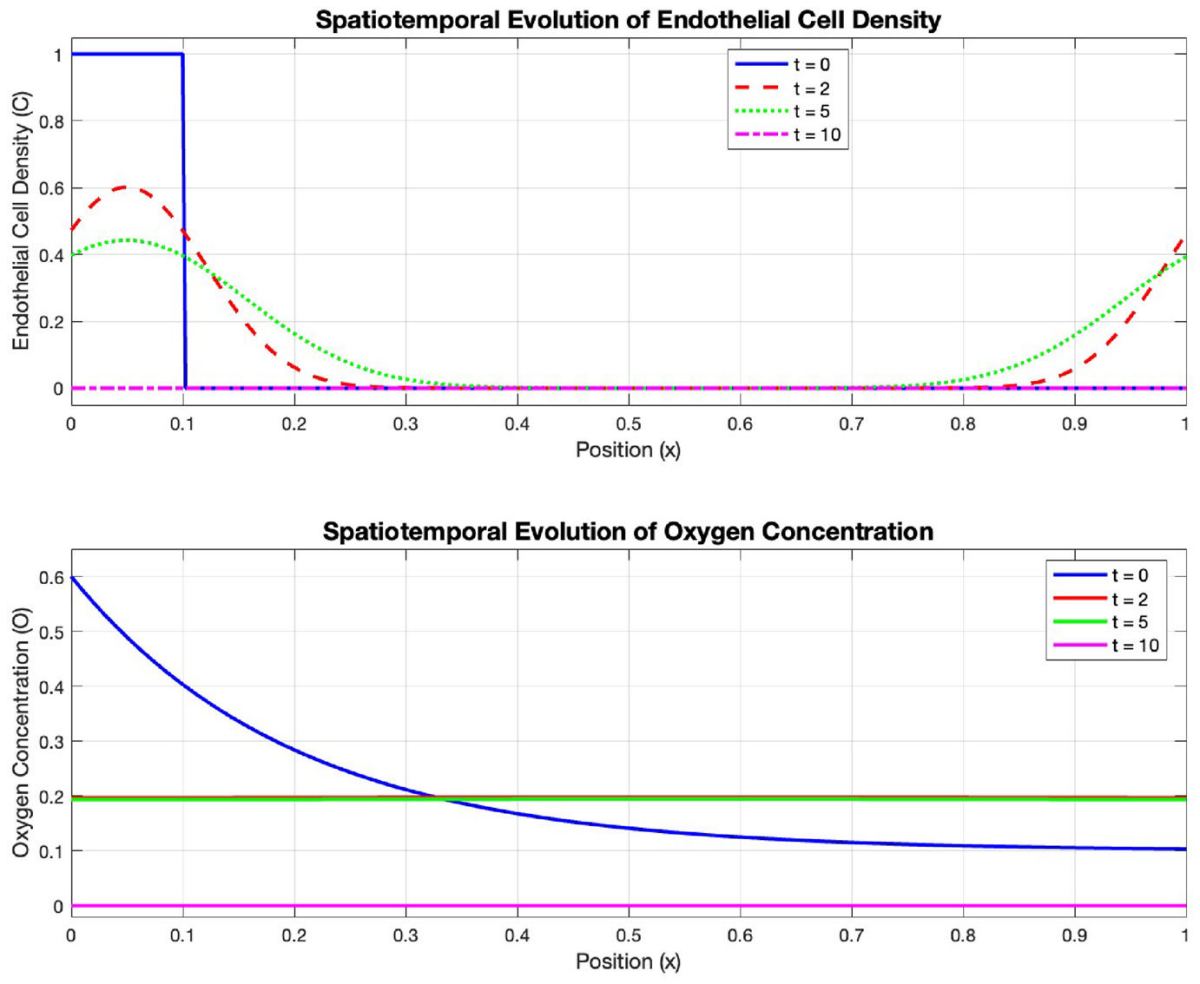
Fig. 1 Spatiotemporal evolution of endothelial cell density *C*(*x*, *t*) (top) and oxygen concentration *O*(*x*, *t*) (bottom) at $t = 0$ (solid blue), 2 (dashed red), 5 (dotted green), 10 (dash-dot magenta). Parent vessel at $x = 0$, tumor at $x = 1$

**Fig. 2:**
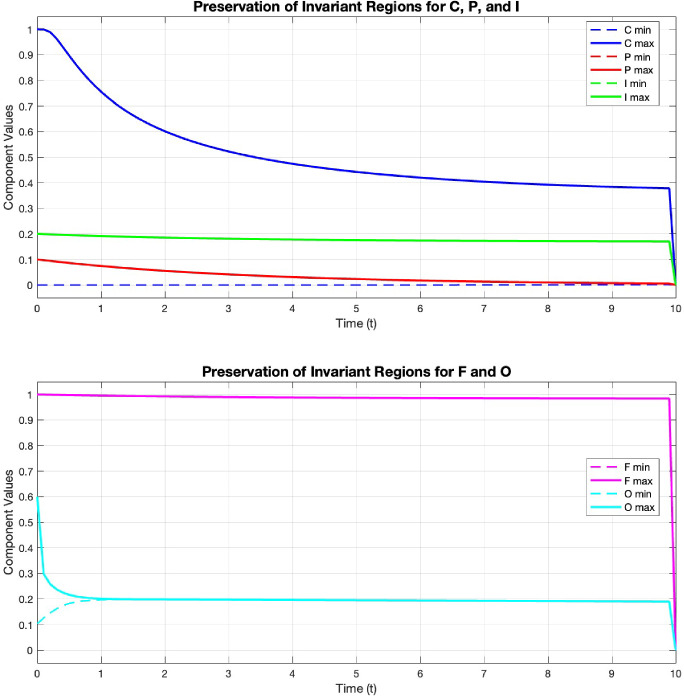
Evolution of minimum and maximum values for extracellular matrix F and oxygen concentration O, demonstrating preservation of invariant regions established in Theorem 6. The bounds remain within their theoretical limits throughout the simulation

The computational domain $\Omega = [0, 1]$ represents tissue between a parent vessel (at $x = 0$) and a tumor (at $x = 1$). The initial conditions are Figure 1:

$$\begin{aligned} C(x,0)&= {\left\{ \begin{array}{ll} 1, & 0 \le x \le 0.1, \\ 0, & 0.1 < x \le 1, \end{array}\right. } \\ P(x,0)&= 0.1, \quad I(x,0) = 0.2, \quad F(x,0) = 1, \\ O(x,0)&= 0.5e^{-5x} + 0.1. \end{aligned}$$

These conditions represent endothelial cells initially present only near the parent vessel, with oxygen diffusing from this vessel and gradually decreasing away from it. We use $M = 256$ spatial grid points with time step $\Delta t = 10^{-5}$ to ensure numerical stability. Figure 2 shows the spatiotemporal evolution of the endothelial cell density *C*(*x*, *t*) and oxygen concentration *O*(*x*, *t*) over 10 time units. The simulation captures the key biological processes of tumor angiogenesis: initial migration of endothelial cells toward the tumor in response to the angiogenic factor gradient, proliferation of endothelial cells as they advance forming a traveling wave, degradation of the extracellular matrix ahead of the advancing front, and increased oxygen concentration following vascular formation.

The simulation exhibits biologically coherent behavior with endothelial cells progressively advancing toward the tumor site, driven by the balance of haptotaxis, chemotaxis, and oxygen-dependent proliferation. The oxygen concentration increases accordingly, demonstrating the functional perfusion established by the nascent vascular network.

**Test 3. Parameter Sensitivity Analysis.** In order to understand the biological implications of parameter variations, we examine how key parameters influence vascular pattern formation. Increasing the oxygen diffusion coefficient $d_O$ from 0.05 to 0.2 results in more uniform oxygen distribution, reducing the driving force for angiogenesis and leading to slower, more diffuse vascular growth. Conversely, higher endothelial proliferation rates $k_1 > 0.2$ accelerate vessel formation but may compromise the realistic branching patterns observed experimentally. The haptotaxis parameter $\alpha _1$ critically influences vessel morphology: values below 0.05 produce poorly directed growth, while $\alpha _1 > 0.3$ generates unrealistic straight-line migration toward the tumor. The chemotactic sensitivity $\alpha _2$ provides a regulatory mechanism, stronger inhibitor response ($|\alpha _2| > 0.15$) creates more tortuous, realistic vessel paths by balancing pro- and anti-angiogenic signals. Oxygen-related parameters demonstrate particularly strong coupling effects. The half-saturation constant $K_O$ modulates the transition between hypoxic and normoxic regimes: lower values ($K_O < 0.1$) create sharper oxygen gradients that drive aggressive angiogenesis, while higher values ($K_O > 0.4$) result in more gradual, controlled vessel development consistent with physiological wound healing rather than tumor angiogenesis.

**Test 4. Preservation of Invariant Regions.** Finally, we verify the theoretical results from Theorem 6 concerning the preservation of positivity and boundedness. Using the same physiological parameters and domain as in the angiogenesis simulation, we track the minimum and maximum values of each component over time. Figure 2 shows the evolution of component bounds throughout the simulation.

As predicted by Theorem 6, all components remain within their respective invariant regions:Of particular interest is the behavior near $t = 3$ when the endothelial cell front reaches the tumor region. Despite the rapid changes in all variables during this period, the numerical scheme maintains solution bounds without introducing spurious oscillations or violations of physical constraints. These results confirm that our numerical scheme accurately preserves the qualitative mathematical properties of the continuous model, validating both the proposed theoretical analysis and numerical implementation.

## Conclusions

In this work we presented an extended mathematical framework by incorporating oxygen concentration, a key factor in tumor progression. The proposed model provided a detailed description, supported by a theoretical framework. On the other hand, a numerical scheme which uses method of lines and Runge-Kutta 4-th is introduced to solve the model. Analytical and numerical analyses explore the impact of this apporach. Numerical experiments confirm the reliability of the proposed scheme, preserving essential angiogenic properties.
